# Disruption of the skin, gill, and gut mucosae microbiome of gilthead seabream fingerlings after bacterial infection and antibiotic treatment

**DOI:** 10.1093/femsmc/xtad011

**Published:** 2023-06-02

**Authors:** Daniela Rosado, Paula Canada, Sofia Marques Silva, Nuno Ribeiro, Pedro Diniz, Raquel Xavier

**Affiliations:** S2AQUA – Collaborative Laboratory, Association for a Sustainable and Smart Aquaculture, Avenida Parque Natural da Ria Formosa s/n, 8700-194 Olhão, Portugal; CIIMAR – Interdisciplinary Center of Marine and Environmental Research, University of Porto, Terminal de Cruzeiros de Leixões. Av. General Norton de Matos, 4450-208 Matosinhos, Portugal; CMC – Centro de Maricultura da Calheta, Direcção Regional do Mar, Av. D. Manuel I, nº 7, 9370-135 Calheta, Madeira, Portugal; CIBIO, Centro de Investigação em Biodiversidade e Recursos Genéticos, InBIO Laboratório Associado, Campus de Vairão, Universidade do Porto, R. Padre Armando Quintas 7, 4485-661 Vairão, Portugal; BIOPOLIS Program in Genomics, Biodiversity and Land Planning, CIBIO, Campus de Vairão, R. Padre Armando Quintas 7, 4485-661 Vairão, Portugal; MVAQUA – Serviços Médico Veterinários dedicados a Aquacultura, Av. do Parque de Campismo Lote 24, Fração C, 3840-264 Gafanha da Boa Hora, Portugal; Marismar – Aquicultura Marinha, Lda, Rua do Cabrestante 28, 9000-105 Funchal, Portugal; CIBIO, Centro de Investigação em Biodiversidade e Recursos Genéticos, InBIO Laboratório Associado, Campus de Vairão, Universidade do Porto, R. Padre Armando Quintas 7, 4485-661 Vairão, Portugal; BIOPOLIS Program in Genomics, Biodiversity and Land Planning, CIBIO, Campus de Vairão, R. Padre Armando Quintas 7, 4485-661 Vairão, Portugal

**Keywords:** fish microbiome, fish infection, aquaculture, oxytetracycline, *Sparus aurata*, dysbiosis

## Abstract

The activity of the microbiome of fish mucosae provides functions related to immune response, digestion, or metabolism. Several biotic and abiotic factors help maintaining microbial homeostasis, with disruptions leading to dysbiosis. Diseases and antibiotic administration are known to cause dysbiosis in farmed fish. Pathogen infections greatly affect the production of gilthead seabream, and antibiotic treatment is still frequently required. Here, we employed a 16S rRNA high-throughput metataxonomics approach to characterize changes in the gut, skin, and gill microbiomes occurring due to infection with *Photobacterium damselae* subsp. *piscicida* and subsequent antibiotic treatment with oxytetracycline (OTC), as well as during recovery. Although microbiota response differed between studied tissues, overall changes in composition, diversity, structure, and predicted function were observed in all mucosae. The skin and gill microbiomes of diseased fish became largely dominated by taxa that have been frequently linked to secondary infections, whereas in the gut the genus *Vibrio*, known to include pathogenic bacteria, increased with OTC treatment. The study highlights the negative impacts of disease and antibiotic treatment on the microbiome of farmed fish. Our results also suggest that fish transportation operations may have profound effects on the fish microbiome, but further studies are needed to accurately evaluate their impact.

## Teaser

Gut, but mostly skin and gills microbiomes are altered in response to Phdp infection and oxytetraciclin treatment, not recovering even after 500º.day

## Introduction

In fish, the microbiome associated with mucosal tissues forms an integrative part of the immune system (Kelly and Salinas 2017), supporting vital physiological functions such as immune response (de Bruijn et al. 2018), and digestion (Liu et al. 2016) or metabolism (Tyagi et al. 2019). Similarly to other vertebrates, fish-associated microbiota can be both modulated by host intrinsic factors, such as age (Rosado et al. 2021a), and by environmental factors, such as temperature (Rosado et al. 2021c, Horlick et al. 2020), salinity (Dulski et al. 2020), dissolved CO_2_ and pH (Fonseca et al. 2019). Changes to these factors are known to alter microbial dynamics, potentially leading to microbial imbalance (dysbiosis) and affecting its function (de Bruijn et al. 2018). These changes can exacerbate the abundance of opportunistic and pathogenic bacteria, which are known to usually colonize the healthy mucosa of fish, increasing disease susceptibility (e.g. Hess et al. 2015, Legrand et al. 2018). Fish mucosal microbial communities are in constant contact with the surrounding water with some exchange of microbes (Gomez and Primm 2021, Sehnal et al. 2021); nevertheless, several studies have demonstrated that the water influence is minor and that fish mucosae are highly selective regarding their microbiome (e.g. Leonard et al. 2014, Carlson et al. 2017, Krotman et al. 2020). However, for the European seabass, skin microbiota was observed to be more permeable to water microbiota, when compared to gill microbiota (Rosado et al. 2021c).

In fish farms, microbial dysbiosis appears to be frequent (Rosado et al. 2021c) and often associated with poor zootechnical conditions, such as low oxygen levels, high ammonia concentrations or suboptimal pH, and host stress (Boutin et al. 2013, Sylvain et al. 2016, Qi et al. 2017, Uren Webster et al. 2018). Recently, an increasing number of studies focusing on the effects of common diseases and chemical treatments in the microbiome of farmed fish have brought to light some interesting insights (e.g. Rosado et al. 2021b, Le Luyer et al. 2021). The results of such studies are generally complex and likely dependent on the target species and studied mucosae, as well as dose-dependent in the case of antimicrobials (e.g. Almeida et al. 2019a, Rosado et al. 2019, Choudhury et al. 2021, Slinger et al. 2021). However, an increase in opportunistic pathogens seems to frequently occur after disease or antibiotic treatment (e.g. Almeida et al. 2019b, Zhang et al. 2018, Slinger et al. 2021). Additionally, in the case of broad-spectrum tetracycline antibiotics (which are extensively used for veterinary purposes; Daghrir and Drogui 2013), significant impacts on microbial community structure (i.e. beta-diversity) were reported for zebrafish (López Nadal et al. 2018, Almeida et al. 2019a, Zhou et al. 2018, 2019b), Atlantic salmon (Navarrete et al. 2008, Slinger et al. 2021), and olive flounder (Kim et al. 2019). Exceptions to this pattern were only reported in a minority of studies and were suggested to be related to existing antibiotic resistance amongst the studied microbiota (Kim et al. 2019, Payne et al. 2021).

Gilthead seabream *Sparus aurata* is one of the most important reared species in the Mediterranean and Atlantic regions (FAO 2018). The production of this species is greatly affected by infectious diseases that can account for losses of up to 40% of the entire fish stock (Lane et al. 2014). Although vaccines and several pre- and probiotics have been tested and confirmed to increase overall fitness and immunity in gilthead seabream (e.g. Cordero et al. 2015, Guardiola et al. 2017, Rimoldi et al. 2020), disease control strategies resorting to antibiotics are still required (Rigos et al. 2021). A large part of microbiome studies in gilthead seabream has focused on the gut commensal communities and how they are affected by diet (e.g. Dimitroglou et al. 2010, Rimoldi et al. 2018, Sanches-Fernandes et al. 2021). In this species, some elements of the gut microbiota were shown to have inhibitory activity against some of the main bacterial pathogens affecting seabream, such as *Vibrio anguillarum, Photobacterium damselae* subsp. *piscicida*, and *Pseudomonas anguilliseptica* (Mancuso et al. 2015). Such research has, therefore, illuminated the importance of gut microbiota for maintaining microbiome homeostasis in this species. However, to the best of our knowledge, there are no studies on the effects of disease or antibiotics on the microbiome of gilthead seabream.

The goal of the present work was to characterize the effects of a disease outbreak and subsequent antibiotic treatment on the microbial dynamics of the skin, gill, and gut microbiome of farmed gilthead seabream fingerlings. Specifically, we used a 16S rRNA high-throughput metataxonomics approach to describe the composition, diversity, structure, and predicted function of such microbial communities; (1) prior to and (2) during the onset of infection with *P. damselae* subsp. *piscicida*, (3) during antibiotic treatment with oxytetracycline (OTC), and (4) at two different time points during recovery.

## Materials and methods

### Experimental design and sample collection

All procedures implying fish handling and sampling were performed by a trained scientist and following the European Directive 2010/63/EU of European Parliament and of the Council of European Union on the protection of animals used for scientific purposes.

The studied fish belonged to a batch of gilthead seabream (*S. aurata*) juveniles (2.8±0.7 g, initial weight; 6.4±0.5 cm, initial total length) that had been purchased from a commercial hatchery in France by a fish farm in Madeira Island to be outgrown in sea cages. Sampled fish belonged to the same cohort, thus with a common zootechnical and clinical history. Fish were killed by a blow to the head and decapitation as a method conditionally accepted by the European Directive PE-CONS 37/10 and the Portuguese Legislation (December 113/2013) to avoid any possible effects of anesthesia on skin and gill microbiome disruption. Each fish was handled using sterilized material.

Skin samples were collected using tubed sterile dry swabs (Medical Wire & Equipment, UK) and by swabbing several times along the right upper lateral part of the fish from head to tail. Gill and gut samples were obtained by excising the right filaments of the second and third arches and the portion between 6 and 3 mm anterior to the rectal sphincter, respectively. Swabs and tissue samples were immediately stored at −20°C until transported on dry ice to the CIBIO-InBIO laboratory by airmail, where they were kept at −80°C until further processing.

Samples were collected in the Autumn, between 28 September and 26 November 2020, encompassing six sampling dates. The initial sampling point occurred upon fish arrival to Madeira Island, after a 5-day transportation from France. Sampling took place after water change in transportation tanks and before transference to the sea-cages (28 September). Samples from this day were categorized as “arrived” (ARV group, *N* = 10). Following arrival and during nine consecutive days, mortality occurred in the sea-cage (> 1%/day), although fish did not present external signs of disease. A total of 2 weeks after arrival (12 October) fish mortality was reduced to < 0.3%/day. Samples were collected on this day and categorized as “asymptomatic” (ASY group, *N* = 8). Mortality suddenly increased on 19 October and peaked on 21 October (∼4.7% of the sea-cage population, corresponding to 12 000 dead fish). On the morning of 22 October, samples from live fish were collected and this group was categorized as “diseased” (DIS group, *N* = 8). Bacterial isolates from the spleen and kidney of infected fish were collected and *P. damselae* spp *piscicida* was identified as the etiological agent of infection via API 20E biochemical test. Results of the API 20E test showed small colonies with tiny dots morphology, streak growth, with no hemolysis, negative for gram coloration, and positive for catalase, oxidase, and motility. Additionally, using antibiogram disc diffusion, colonies were shown to be susceptible to OTC, with a disc diameter of 36 mm. A 10-day antibiotic treatment with OTC started in the afternoon of 22 October, being administered at 15 kg of active component/ton of commercial feed at a feeding rate of 1% until 31 October. On the 7th day under antibiotic treatment, fish microbiomes were sampled again (27 October) and samples were categorized as “under treatment” (TRT group, *N* = 8). During treatment, mortality remained high (1%–3%/day). On 2 November, 2 days after the end of OTC treatment, fish microbiomes were sampled again and samples were categorized as “recovery1” (RCV1, *N* = 8). From this day one mortality in the sea-cage was < 0.1%/day. On 26 November, 26 days after the last day of antibiotic administration, fish microbiomes were sampled for the last time and samples were categorized as “recovery2” (RCV2, *N* = 8). This final sampling date was chosen as it corresponded to >500^o^-days, after which fish would be considered safe for consumption, according to the Portuguese and European legislation (decree-law nº314/2009, under article 78), as any antibiotic residues in the muscle would be under legal limits. This metric is calculated based on temperature times the number of days after administering the last dose of medication. Thus, “recovery1” date would correspond to 43^o^-days and “recovery2” date to 528^o^-days.

### DNA extraction and 16S rRNA sequencing

Total DNA from 145 samples (50 skin, 50 gills, and 45 guts) and six controls (DNA extraction kit controls) was extracted using the PowerSoil DNA Isolation Kit (QIAGEN, the Netherlands), following the manufacturer’s protocol. DNA extractions were shipped in dry ice to the University of Michigan Medical School (USA) for amplification and sequencing according to the protocol of Kozich et al. (2013). The V4 (∼250 bp) hypervariable region of the bacterial 16S rRNA gene was amplified for all samples using the primers developed by Caporaso et al. (2011). Each sample plus PCR blanks were sequenced on a single run of the Illumina MiSeq platform.

In total, 3 124 336 partial 16S rRNA gene sequences were retrieved for all seabream individuals. A total of 10 amplicon sequence variants (ASVs) present in negative controls (extraction kit and PCR) were removed from downstream analysis. After the removal of contaminants and nonbacterial sequences, 1578 ASVs (785 950 sequences), 379 ASVs (815 062 sequences), and 811 ASVs (235 797 sequences) were assigned to the skin, gill, and gut microbiota of the seabream, respectively. Microbial taxa showing a mean relative proportion ≥ 5% in each group within tissue were considered as part of the most abundant taxa in the respective microbiota.

### Data and statistical analysis

Raw FASTQ files were denoised using the DADA2 pipeline in R v4.0.2. (Callahan et al. 2016). A table containing ASVs was constructed and normalized using the negative binomial distribution (McMurdie and Holmes 2014). Taxonomic inferences were done against the SILVA (138 release) reference database (Quast et al. 2013). A midpoint rooted tree of ASVs was estimated using the Quantitative Insights Into Microbial Ecology 2 package (QIIME2; release 2020.11; Bolyen et al. 2019). The core microbiota was assessed at the ASV level for each group (i.e. sampling date) separately. An ASV was considered part of the core microbiota if present in 100% of the samples from each group.

All analyses were performed in R-studio v4.0.2. Microbial alpha-diversity was estimated at the ASV level using Shannon and Faith’s phylogenetic (PD) diversity indices as implemented in the R *phyloseq* and *picante* packages (Webb et al. 2008, McMurdie and Holmes 2013). Additionally, Pielou’s evenness was calculated at the ASV level as implemented in the R *microbiome* package (Lahti and Shetty 2018). Microbiome structure (beta-diversity) was also estimated at the ASV level using phylogenetic UniFrac (weighted and unweighted) and Bray–Curtis distances using the *phyloseq* package (McMurdie and Holmes 2013). Variation in microbial alpha-diversity between groups was assessed using linear models (lm) as implemented in the R *stats* package (R Core Team 2012), while variation in microbial beta-diversity was assessed using permutational multivariate analysis of variance (PERMANOVA) using the *adonis* function of the R *vegan* package (Oksanen et al. 2008). The effect of individual’s size and weight on microbial diversity (for both alpha and beta-diversity measures) were initially tested and showed no significant impact (*P* > 0.05; results not shown); thus, these variables were not included in any subsequent analyses. The dissimilarity between samples was visually assessed through principal coordinates analysis (PCoA).

Predicted bacterial metabolic functions were estimated using the metagenomic Phylogenetic Investigation of Communities by Reconstruction of Unobserved States software (PICRUSt2) embedded in QIIME2 and applying a weighted nearest sequenced taxon index (NSTI) cutoff of 0.03 (Bolyen et al. 2019, Douglas et al. 2019). Predicted functions were collapsed using the Kyoto Encyclopedia of Genes and Genomes (KEGG) pathway metadata (Kanehisa et al. 2019). Linear discriminant analysis (LDA) in LEfSe was used to identify differentially abundant metabolic pathways in the microbiota of each tissue using group as a class, a *P*-value cut-off of .05 and a LDA effect size cutoff of 2 (Segata et al. 2011).

## Results

### Microbial diversity

**Figure 1.**
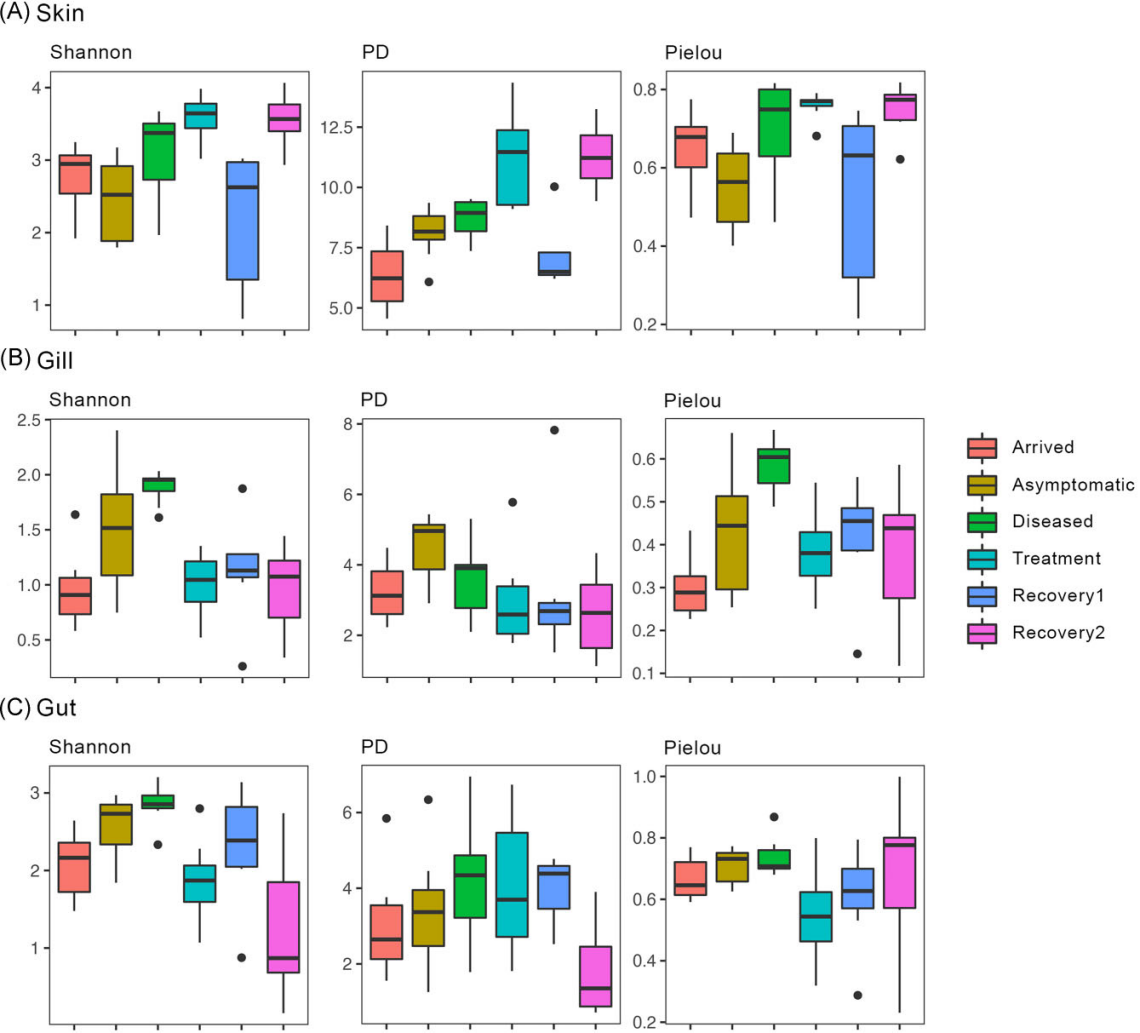
Mean values and standard deviations of Shannon index, Faith’s PD, and Pielou’s evenness plotted for the skin (A), gill (B), and gut (C) microbiome of the seabream *S. aurata* across the different groups. The dots represent sample outliers.

Generally, there were no significant differences in alpha-diversity estimates of the microbial communities of any tissues between the Arrived and Asymptomatic groups (Table 1). The impact of disease on microbial alpha-diversity was not significant for any of the tissues (*P* ≥ .1; Table 1). In the skin, Faith’s PD significantly increased with treatment (*P* = .004; Table 1, Fig. 1), denoting an increase in PD between the Diseased and Under-treatment groups in this tissue. On the other hand, Pielou’s decreased significantly in the gill (*P* = .01; Table 1, Fig. 1), while the Shannon index significantly decreased both in the gill and gut (*P* ≤ .02; Table 1, Fig. 1), expressing a decrease in species richness and diversity in these tissues due to antibiotic administration. Significant differences in alpha-diversity during recovery (between Treatment and Recovery1, and Rocovery1 and Recovery2) were only observed in the skin (*P* ≤ .01, Table 1), which decreased and then increased (Fig. 1). Finally, all estimates of alpha-diversity of the skin microbiome significantly increased between the Asymptomatic and Recovery2 groups (*P* ≤ .02; Table 1, Fig. 1).

**Table 1.** Microbial diversity comparisons for the skin, gill, and gut of the seabream *S. aurata* across all samples (overall) and between groups (ARV: Arrived; ASY: Asymptomatic; DIS: Diseased; TRT: Treatment; RCV1: Recovery1; and RCV2: Recovery2). For each anova test (alpha-diversity), we report the *F-*value (overall) and significance (*P-*value, overall and pairwise), and for each PERMANOVA test (beta-diversity), we report the *R*^2^ statistics and significance. Significant differences (*P* ≤ .05) are indicated in bold.

Tissue	Metric	Overall	ARV/ASY	ASY/DIS	DIS/TRT	TRT/RCV1	RCV1/RCV2	ASY/RCV1	ASY/RCV2
Skin	Shannon	**8.3 (1^–^^5^)**	0.8	0.2	0.5	**0.0002**	**0.0002**	0.9	**0.004**
	Faith’s PD	**20.4 (2^–^^10^)**	0.1	0.9	**0.004**	**0.000 002**	**0.000 003**	0.7	**0.0004**
	Pielou	**5.4 (0.001**)	0.5	0.1	0.9	**0.01**	**0.01**	0.9	**0.02**
	Unweighted UniFrac	**0.3 (9^–^^5^)**	**0.6 (0.02)**	**0.5 (0.02)**	**0.5 (0.02)**	**0.3 (0.02)**	**0.4 (0.02)**	**0.4 (0.02)**	**0.5 (0.03)**
	Weighted UniFrac	**0.3 (0.001)**	**0.4 (0.03)**	0.1 (1)	0.1 (1)	0.3 (0.5)	0.3 (0.2)	0.4 (0.1)	0.4 (0.1)
	Bray–Curtis	**0.5 (9^–^^5^)**	**0.8 (0.02)**	**0.6 (0.02)**	**0.6 (0.02)**	**0.5 (0.02)**	**0.5 (0.02)**	**0.6 (0.02)**	**0.7 (0.02)**
Gill	Shannon	**8.6 (9^–^^6^)**	**0.03**	0.3	**0.0003**	0.9	0.9	0.4	0.1
	Faith’s PD	21. (0.1)	0.3	0.7	0.9	0.9	0.9	0.3	0.1
	Pielou	**6.5 (0.0001)**	0.1	0.1	**0.01**	0.9	0.9	0.9	0.9
	Unweighted UniFrac	**0.5 (9^–^^5^)**	**0.7 (0.02)**	**0.3 (0.02)**	**0.3 (0.05)**	0.1 (1)	0.1 (1)	**0.5 (0.02)**	**0.5 (0.02)**
	Weighted UniFrac	**0.9 (9^–^^5^)**	**0.9 (0.02)**	**0.7 (0.02)**	**0.5 (0.03)**	0.01 (1)	**0.7 (0.02)**	**0.6 (0.02)**	**0.8 (0.02)**
	Bray–Curtis	**0.6 (9^–^^5^)**	**0.9 (0.02)**	**0.5 (0.02)**	**0.4 (0.03)**	0.03 (1)	**0.6 (0.03)**	0.9 (0.2)	**0.6 (0.02)**
Gut	Shannon	**6.8 (0.0001)**	0.6	0.9	**0.02**	0.7	**0.02**	0.9	**0.002**
	Faith’s PD	**2.7 (0.04)**	0.9	0.9	0.9	0.9	0.1	0.9	0.3
	Pielou	1.7 (0.2)	0.9	0.9	0.1	0.9	0.9	0.8	0.9
	Unweighted UniFrac	**0.3 (9^–^^5^)**	**0.6 (0.02)**	0.1 (1)	0.2 (0.8)	0.2 (0.6)	**0.5 (0.02)**	0.1 (1)	**0.4 (0.03)**
	Weighted UniFrac	**0.4 (9^–^^5^)**	**0.8 (0.02)**	0.1 (1)	**0.6 (0.02)**	0.1 (1)	0.3 (0.1)	0.5 (0.2)	**0.7 (0.02)**
	Bray–Curtis	**0.3 (9^–^^5^)**	**0.8 (0.02)**	0.04 (1)	**0.6 (0.02)**	0.3 (0.1)	**0.5 (0.05)**	0.2 (1)	**0.8 (0.02)**

Beta-diversity was significantly impacted by the disease in the skin and gill (*P* ≤ .02; Table 1) and by treatment in all tissues (*P* ≤ .05; Table 1). Additionally, the effects of the recovery were significantly noticed, first in the skin (between the Under-treatment and Recovery1 groups; *P* ≤ .02; Table 1), and then in all tissues (between the Recovery1 and Recovery2 groups; *P* ≤ .05; Table 1). Additionally, the Asymptomatic group showed significant differences in beta-diversity estimates in comparison with the Recovery1 group in the skin and gill (*P* ≤ .02; Table 1), as well as in comparison with the Recovery2 group in all tissues (*P* ≤ .03; Table 1).

**Figure 2.**
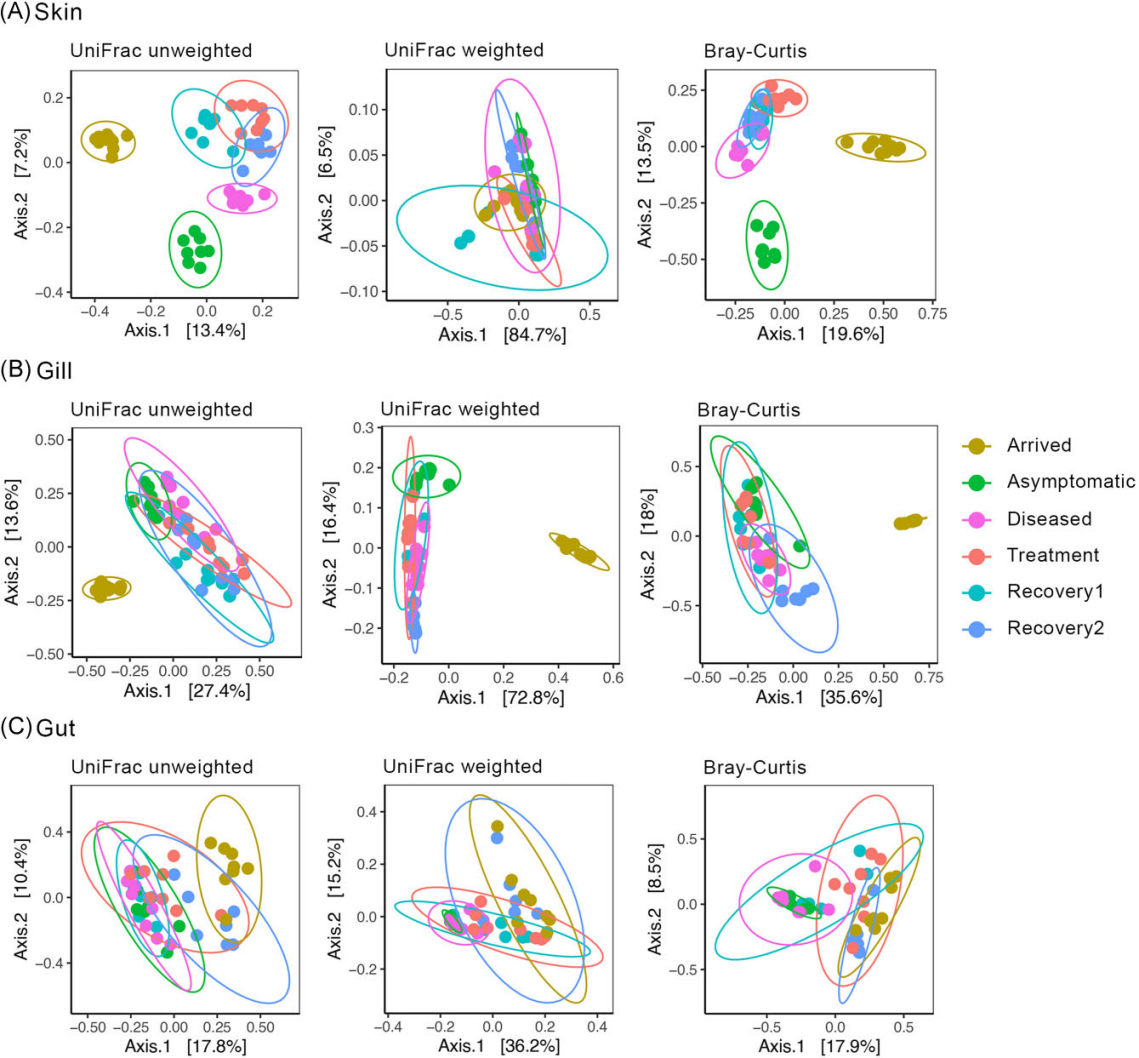
Beta-diversity represented by PCoA plots computed with unweighted and weighted Unifrac indexes and Bray–Curtis distance for the skin (A), gill (B), and gut (C) microbiome of the seabream *S. aurata* across the different groups. Each dot represents a microbiome sample and is colored by group.

PCoA showed clear dissimilarities in the microbial communities of the skin and gill between the Arrived and the remaining groups along the first axis, which explained between 13.4%–19.6% and 27.4%–72.8% of the variation occurring in the skin and gill, respectively (Fig. 2). The PCoA of unweighted UniFrac and Bray–Curtis distances also showed a clear separation of the skin microbiota between the Asymptomatic and Diseased + Treatment + Recovery1 + Recovery2 groups along the second axis of the PCoA (Fig. 2). PCoAs depicting dissimilarities in the gut microbiota showed no apparent distinction between sampling dates, although there was less variation between Asymptomatic fish when compared to fish in the other groups (Fig. 2).

### Microbial taxonomic characterization

**Figure 3.**
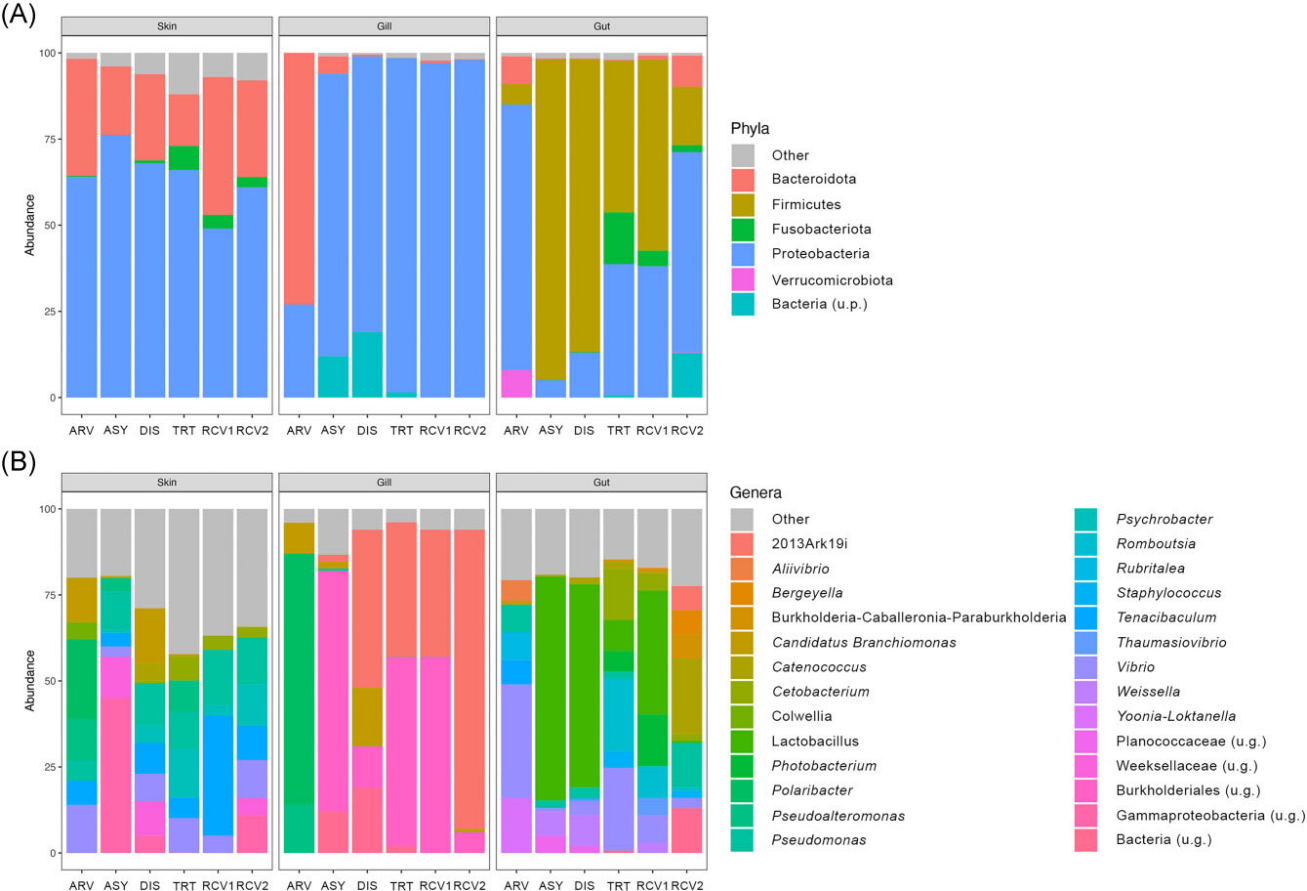
Relative frequency of the most abundant (≥5%) phyla (A) and genera (B) recovered from the seabream *S. aurata* microbiome across groups (ASY—Asymptomatic, HLT—Healthy, DIS—Diseased, TRT—Treatment, RCV1–Recovery1, and RCV2–Recovery 2). Unknown phyla and genera are identified as u.p. and u.g., respectively.

Changes to the microbiome taxonomic composition of the three assessed tissues occurred throughout the study, both at the genus and phylum levels. Initially (i.e. in the Arrived groups), Bacteroidota and Proteobacteria were the dominant phyla in the skin and gill microbiome, whereas Bacteroidota, Proteobacteria, Firmicutes, and Verrucomicrobiota were highly abundant in the gut (Fig. 3; Table S1, Supporting Information). The genera *Tenacibaculum* (13 ± 12%) and *Vibrio* (10 ± 3%) were highly abundant in the skin microbiome, except in the Asymptomatic group, while the most abundant genera in the gill and gut microbiome varied through time (Fig. 3; Table S1, Supporting Information).

After 14 days upon arrival (i.e, Asymptomatic group), the microbiome of the three tissues had suffered a turnover, with Bacteroidota decreasing by 2-, 15-, and 27-fold in the skin, gill and gut, respectively (Fig. 3; Table S1, Supporting Information). Concomitantly, Proteobacteria increased by 1.2- and 3-fold in the skin and gill microbiome, respectively; while in the gut a decrease of 15-fold was observed (Fig. 3; Table S1, Supporting Information). Additionally, in the gut, the abundance of Firmicutes increased by 16-fold and Verrucomicrobiota was no longer present in the Asymptomatic group (Fig. 3; Table S1, Supporting Information). The most abundant genera in the microbiome of the Arrived group in the three tissues were almost completely replaced by other abundant genera in the Asymptomatic group, with only *Pseudomonas* spp. maintaining a high abundance in the skin throughout the study (12 ± 3%; Fig. 3; Table S1, Supporting Information).

In the gill microbiome of the Diseased group, the abundance of Bacteroidota decreased by 10-fold, while in the gut microbiome the abundance of Proteobacteria increased and Firmicutes decreased (Fig. 3; Table S1, Supporting Information). The skin microbiome of infected fish (Diseased group) were dominated by *Candidatus Branchiomonas* (16%), *Pseudomonas* (12%), one genus from the family Weeksellaceae (10%), *Tenacibaculum* (9%), *Vibrio* (8%), *Psychrobacter* (5%), *Catenococcus* (5%), and one genus from the class Gammaproteobacteria (5%) (Fig. 3; Table S1, Supporting Information). The gill microbiome of these fish was largely dominated by 2013Ark19i (46%), *Candidatus Branchiomonas* (17%), an unidentified genus (19%) from an unidentified bacteria phylum, and a genus from the order Burkholderiales (12%); and the gut microbiome of infected fish were dominated by *Lactobacillus* (59%) and *Weissella* (9%) (Fig. 3; Table S1, Supporting Information).

Antibiotic administration induced an increase in the abundance of Proteobacteria in the gill and gut, Fusobacteria in the gut, and a decrease of Firmicutes in the gut (Fig. 3; Table S1, Supporting Information). In the skin microbiome, *Pseudomonas, Vibrio*, and *Tenacibaculum, Psychrobacter, Pseudoalteromonas*, and *Cetobacterium* were highly abundant in the Treatment group (Fig. 3; Table S1, Supporting Information). Also in the Treatment group, the gill microbiome was dominated by 2013Ark19i and by a genus from the order Burkholderiales (Fig. 3; Table S1, Supporting Information). In the gut microbiome, the most abundant genera in the Treatment group were *Lactobacillus, Vibrio, Romboutsia, Photobacterium, Cetobacterium*, and *Staphylococcus* (Fig. 3; Table S1, Supporting Information).

During recovery, Bacteroidota increased in abundance in the gut (Recovery2 group), slightly surpassing the abundance observed initially (Arrived group), while the abundance of Proteobacteria increased in the Recovery2 group and the abundance of Fusobacteria increased in the Recovery 1 group (Fig.   3; Table S1, Supporting Information). In the skin microbiome, *Pseudomonas, Vibrio*, and *Tenacibaculum* were again highly abundant in the Recovery1 and Recovery2 groups, while *Psychrobacter*, a genus belonging to the Gammaproteobacteria class and a genus from the Weeksellaceae family were highly abundant but only in the Recovery2 group (Fig.   3; Table S1, Supporting Information). In the gill, the highly abundant genera found in the Under-treatment group were also observed throughout the remainder sampling dates (Fig.   3; Table S1, Supporting Information). In the gut microbiome, the most abundant genera in the Recovery1 group were the same as in the Under-treatment group, with the replacement of *Staphylococcus* for *Thaumasiovibrio* (Fig. 3; Table S1, Supporting Information). Lastly, in the gut of Recovery2 group, a complete turnover of dominant genera was observed, with the gut microbiome being mainly comprised of *Catenococcus, Pseudomonas*, an unidentified genus from an unidentified phylum, *Bergeyella*, 2013Ark19i, and *Burkholderia–Caballeronia–Paraburkholderia* (Fig. 3; Table S1, Supporting Information).

### Core microbiome

There were a total of 57, 17, and 6 core ASVs across all analyzed groups of the skin, gill, and gut microbiome, respectively (Table S2, Supporting Information). The percentage of core ASV between the Arrived and Asymptomatic groups decreased in the skin (7%–4%) and in the gill (5%–4%), while no core ASVs were observed in the Arrived group of the gut (Table S2, Supporting Information). With infection, the percentage of core ASVs was maintained in the skin (4%), increased in the gill (8%), and decreased in the gut (2%–1%; Table S2, Supporting Information). After the administration of the antibiotic, this percentage was also maintained in the skin (4%) and decreased in the gill (7%) and gut (0%; Table S2, Supporting Information). During recovery, a decrease (Recovery1, 3%) and then an increase (Recovery2, 5%) of the percentage of core ASVs was observed in the skin, while a decrease (4%) was observed in the gill in the Recovery1, a percentage that was maintained in the Recovery2 group (Table S2, Supporting Information). In the gut, the percentage of core ASVs was maintained in the Recovery1 group, while no core ASVs were observed in the Recovery2 group (Table S2, Supporting Information).

It is important to notice that, within the skin core ASVs, there were nine belonging to potentially pathogenic genera, namely *Acinetobacter* (Diseased and Recovery1 groups), *Janthinobacterium* (Asymptomatic, Diseased, Recovery1, and Recovery2 groups), *Polaribacter* (Arrived group), *Pseudomonas* (Asymptomatic group), *Stenotrophomonas* (Arrived, Asymptomatic, Diseased, and Recovery 1 groups), *Tenacibaculum* (Arrived, Asymptomatic, Diseased, and Recovery 1 groups), and *Vibrio* (Arrived, Asymptomatic, Treatment, and Recovery2 groups); and two pathogenic species *Leucothrix mucor* (Arrived group) and *Tenacibaculum dicentrarchi* (Recovery2 group) (Table S2, Supporting Information). Likewise, some ASVs belonging to genera known to harbor potentially pathogenic strains were recovered from the gill core microbiome, including 2013Ark19i (Asymptomatic, Diseased, Treatment, and Recovery2 groups), *Candidatus Branchiomonas* (Arrived, Diseased, and Recovery2 groups), and *Vibrio* (Arrived group) (Table S2, Supporting Information).

### Microbial predicted function

**Figure 4.**
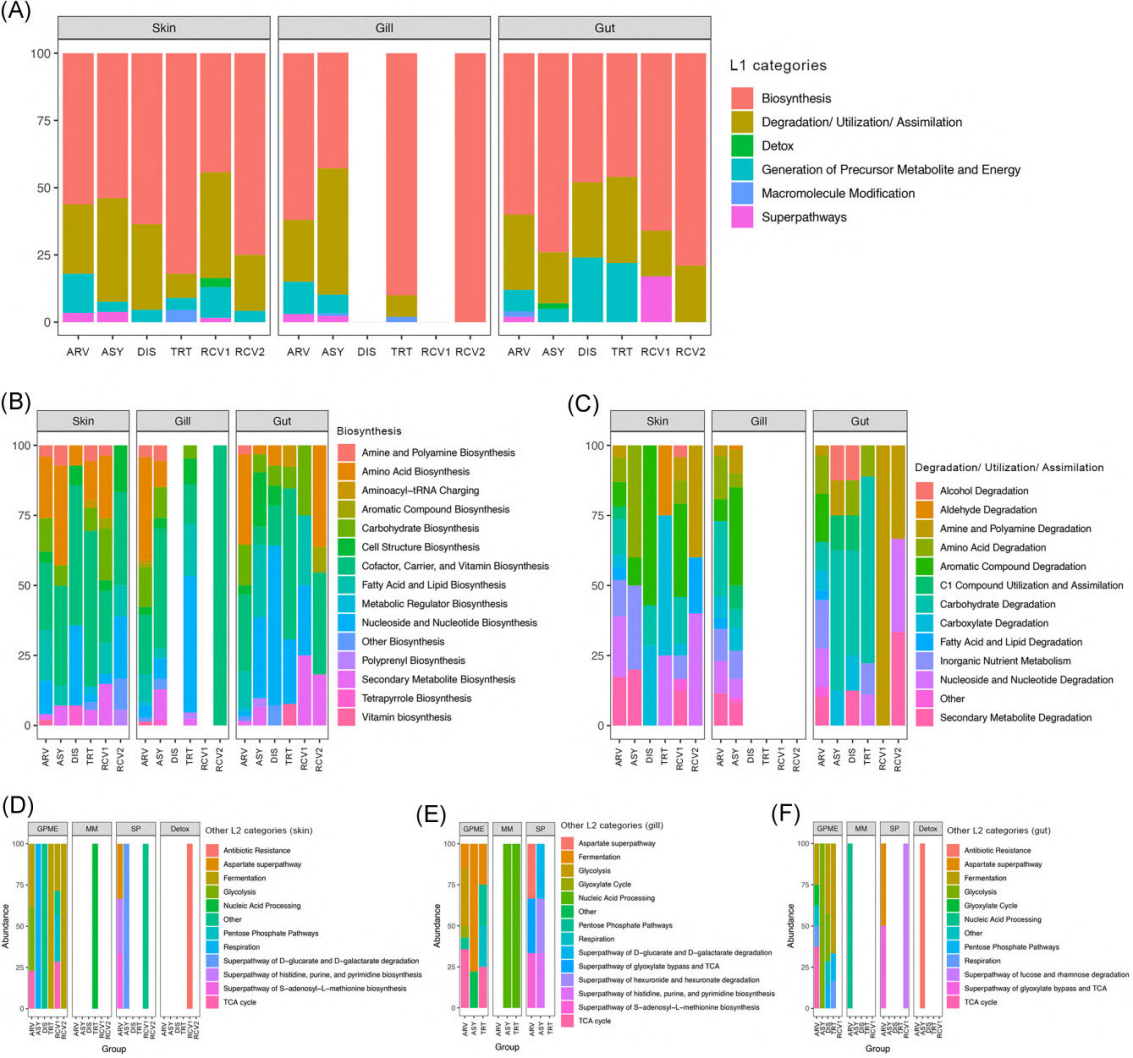
Relative frequency of the differently enriched predicted pathways in the gill, gut, and skin microbiome of the seabream *S. aurata* across groups (ASY—Asymptomatic, HLT—Healthy, DIS—Diseased, TRT—Treatment, RCV1–Recovery1, and RCV2–Recovery 2). Enriched predicted pathways are grouped by broader (A) or smaller (B)–(F) KEGG categories. Smaller categories are divided into biosynthesis (B), degradation/utilization/assimilation (C), and other less-enriched categories (D)–(F). Within the latter (GPME—General Precursor Metabolite and Energy, MM—Macromolecule Modification, SP—superpathways, and Detox—Detoxification), barplots are divided by tissue, i.e. skin (D), gill (E), and gut (F).

Overall, there were 449, 399, and 411 KEGG pathways inferred from the skin, gill, and gut microbiota of the seabream, respectively. LDA showed that several predicted pathways were significantly enriched in the Arrived (89, 114, and 103 in the skin, gill, and gut, respectively), Asymptomatic (26, 127, and 42 in the skin, gill, and gut, respectively), Diseased (22 and 29 in skin and gut, respectively), Treatment (44, 48, and 28 in the skin, gill, and gut, respectively), Recovery1 (61 and 6 in the skin and gut, respectively), and Recovery2 (24, 14, and 4 in the skin, gill, and gut, respectively) groups (Table S3, Supporting Information). It is important to note that there were no significantly enriched pathways in the gill microbiome of the Diseased and Recovery1 groups (Fig. 4; Table S3, Supporting Information). Overall, there were more enriched pathways in the Arrived (skin and gut) and Asymptomatic (gill) fish compared to the other groups. Generally, in all mucosae, there was always a higher percentage of pathways related to biosynthesis, followed by pathways related to degradation/utilization/assimilation (Fig. 4; Table S3, Supporting Information). To a lesser extent, an enrichment of pathways related to generation of precursor metabolite and energy was also observed in almost all groups, while pathways related to macromolecule modification and superpathways were enriched in only two groups per tissue on average (Fig. 4; Table S3, Supporting Information). Pathways related to detoxification were only enriched in the Asymptomatic and Recovery1 groups in the gut and skin, respectively (Fig. 4; Table S3, Supporting Information). At a finer scale, the frequency of predicted pathways of the microbiome of the three tissues varied greatly between groups (Fig. 4; Table S3, Supporting Information).

Enriched pathways between the Arrived and Asymptomatic groups decreased in the skin, while in the gill enriched metabolic pathways related to Biosynthesis decreased and those related to degradation/utilization/assimilation increased (Fig. 4; Table S3, Supporting Information). In the skin, the number of enriched metabolic pathways remained stable when the disease occurred, while in the Under-treatment group the number of enriched pathways doubled, mainly due to a more than 2-fold increase in biosynthesis-related pathways (Fig.   4; Table S3, Supporting Information). Compared to the other two tissues, the metabolic function of the gill microbiota seems to be the most affected by disease and treatment; however, there were no enriched degradation/utilization/assimilation pathways in the gill during treatment (Fig. 4; Table S3, Supporting Information). On the contrary, disease and antibiotic treatment seemed to have a smaller effect on the predicted metabolic function of the gut, even though a sharp decrease in the total number of metabolic pathways between the Arrived and other groups was observed (Fig. 4; Table S3, Supporting Information).

In the skin, degradation/utilization/assimilation increased in the Recovery1 group and decreased in Recovery2. Detoxification pathways related to antimicrobial resistance were enriched in the Recovery1 group. Comparing the Asymptomatic and Recovery2 groups, although there was a decrease in the total number of enriched metabolic pathways, the proportion between biosynthesis and degradation/utilization/assimilation pathways (the two main pathways found herein) was similar between the two groups. Additionally, in both groups, six different classes of biosynthesis pathways were found, and although cofactor, carrier and vitamin biosynthesis and fatty acid and lipid biosynthesis were the only ones that were common to the two groups, this shows that the metabolic potential of skin microbiota at the end of recovery is high and somewhat comparable to the Asymptomatic group. In the gill, only four biosynthesis pathways were enriched in the Recovery2 group. Compared to the other two tissues, the metabolic function of the gill microbiota seems to be the one with more striking differences between the Asymptomatic and Recovery groups. Regarding the predicted metabolic function of the gut, the number of enriched metabolic pathways decreased 2- to 3-fold during recovery, as predicted functions were less variable as well.

## Discussion

The mucosal tissues of fish are a vital part of the teleost immune system. Being major pathways for pathogen entrance (Merrifield and Ringø 2014), fish mucosae function as fundamental barriers to pathogens (Salinas and Magadán 2017). In the present study, we characterized dysbiosis of the skin, gill, and gut microbiomes of gilthead seabream fingerlings caused by photobacteriousis, a disease induced by *P. damselae* subsp. *piscicida* and subsequent OTC treatment. We also report the recovery trajectory of the microbiotas in these mucosal tissues after the end of a 10-day OTC treatment and until full metabolization of antibiotics.

The main focus of the present work was to assess the effects of infection and antibiotic treatment in the mucosae of the gilthead seabream. However, having analyzed apparently healthy fish from the studied cohort just upon arrival (arrived group) after a 5-day transportation operation, and then after a 14-days acclimation period (asymptomatic group), the striking changes in the mucosal microbial communities between these two initial sampling points raise an unavoidable discussion around the possible effects of transportation. Transportation of live fish between aquaculture facilities is a necessary common practice that may induce physiological stress responses and increase vulnerability to disease (e.g. Pankhurst 2011, Tort 2011, Sampaio and Freire 2016). The negative effects of transport may be linked to nonoptimal water conditions to which fish may be subjected to, as well as to stress induced by very high densities and starvation (Sampaio and Freire 2016, Stevens et al. 2017). In fact, the growth of potentially pathogenic bacteria can be boosted by prolonged exposure to stress (e.g. Boutin et al. 2013, Sylvain et al. 2016, Uren Webster et al. 2020). Although the effects of transportation on the mucosal microbial communities of fish are nearly unexplored, various detrimental consequences have been reported. For example, epitheliocystis outbreaks are typically associated with recent stressful operations such as transportation (Seth-Smith et al. 2016). In the present work, *Candidatus Branchiomonas*, a known agent for epitheliocystis in Atlantic salmon (Mitchell et al. 2013) and gilthead seabream (Seth-Smith et al. 2016), was abundant in the skin and gill of the studied fish upon arrival, but not after acclimation. Additionally, the present results also show more general striking changes in microbial composition, diversity, and predictive function between the first two sampling points, which are indicative of an important microbial disruption. These changes are likely the result of a compound effect of stress associated with transportation and acclimation to a novel environment and zootechnical conditions (e.g. diet and physicochemical water properties).

### Effects of disease on the mucosal microbiome of the gilthead seabream

Fish production intensification has been accompanied by an increase in the occurrence of pathological outbreaks of infectious diseases (FAO 2018). Many studies have established a link between disease and microbial imbalance, or dysbiosis, in fish. For example, changes in the composition, diversity, and predicted function of the mucosal microbiome due to disease caused by bacterial pathogens have been reported for several farmed fish species (e.g. Rosado et al. 2019, Miyake et al. 2020, Le Luyer et al. 2021, 2021b). Even though these are general fingerprints of disease in fish microbiota, reported patterns associated with dysbiosis differ between host species and the etiological agent of disease, as well as on other factors such as studied tissue. In the present study, infection by *P. damselae* subsp. *piscicida* did not significantly affect microbial alpha-diversity in the studied tissues. However, it was possible to observe a turnover of dominant taxa and changes in microbial community structure (i.e. beta-diversity) in the skin and gill of gilthead seabream fingerlings. On the contrary, gut microbial community structure measures remained unaffected by the disease.

Photobacteriousis has been responsible for significant losses in Mediterranean farmed populations of several fish species, including gilthead seabream (e.g. Jesús 2002). Although adults of the gilthead seabream present higher resilience to this disease, mortality in larvae and juvenile populations can reach 100% (Pellizzari et al. 2013). Usually, photobacteriousis causes septicemia as well as internal lesions in the liver, spleen, and kidney, and less commonly external lesions in the head, gills, and skin (Santos et al. 2022). Infection is usually regarded as systemic but histopathological effects are not frequently reported in the gut. In fact, in the present study, the gut microbial communities were less affected than those in the skin or gill.

The present results show a noticeable decrease in enriched predicted metabolic pathways in all three tissues of diseased individuals when compared to asymptomatic fish. The most striking occurred in the gill where there was a total obliteration of enriched functional pathways. Decreases in enriched pathways related to biosynthesis were also noticeable in the gut, but not the skin microbiota. Interestingly, similar microbial functions in response to disease were found in the skin and gut microbiota, where most enriched metabolic pathways were predicted to be related to nucleoside and nucleotide biosynthesis, as well as to degradation pathways, such as carbohydrate (in the gut) and aromatic compounds (in the skin) degradation. Furthermore, pathways related to the generation of precursor metabolites and energy through glycolysis and fermentation were also enriched in the gut. Indeed, the gut microbiome of individuals from the Diseased group was largely dominated by *Lactobacillus* and *Weisella*, both lactic-acid bacteria that use fermentation and with known probiotic activity against pathogens (Hoseinifar et al. 2018, Van Doan et al. 2019). Although, to the best of our knowledge, *P. damselae* subsp. *piscicida* inhibition by *Lactobacillus* and *Weissella* strains has not yet been tested, the high abundance of these taxa in the gut of diseased individuals suggests they could have some role in limiting dysbiosis in this tissue. On the contrary, the skin and gill microbiome of the Diseased group became largely dominated by taxa that have been frequently linked to secondary infections, such as *Pseudomonas* (e.g. Llewellyn et al. 2017, Dong et al. 2019, Rosado et al. 2019), *Vibrio* (e.g. Rosado et al. 2021b, Llewellyn et al. 2017, Le Luyer et al. 2021), *Tenacibaculum* (e.g. Llewellyn et al. 2017, Reid et al. 2017), or *Candidatus Branchiomonas* (Mitchell et al. 2013, Seth-Smith et al. 2016). Such potential deleterious changes could have ultimately exacerbated dysbiosis in these tissues. Secondary infections frequently occur in aquaculture fish and can exacerbate the severity of disease outbreaks (Kotob et al. 2016).

Interestingly, although *Photobacterium* was identified as the primary etiological agent for the observed disease, it was not among the most abundant genera present in the skin, gill, or gut microbiomes of diseased individuals. This result adds to previous studies on microbiotas in the skin of fingerlings and adult European seabass suffering from photobacteriousis (Rosado et al. 2019, 2021b). Although significant alterations to the microbiome occurred in the studied tissues due to the onset of disease, they were likely a result of changes in host homeostasis rather than being caused directly or exclusively by increased abundance of the etiological agent.

### Effects of OTC on the mucosal microbiome of the gilthead seabream

OTC is a broad-spectrum antibiotic extensively used for human therapy, veterinary, and agricultural purposes (Daghrir and Drogui 2013). The impacts of its use on the microbiome have been evaluated in several fish species, including Nile tilapia (Payne et al. 2021), zebrafish (e.g. Almeida et al. 2019a,b,^1^), rainbow trout (Choudhury et al. 2021), olive flounder (Kim et al. 2019), and the European seabass (Rosado et al. 2019). In line with our results, these studies also reported changes to the composition, diversity, and predicted function of the microbial communities of the mucosal tissues. Additionally, some of these studies also reported an enrichment of opportunistic pathogens linked to an increased susceptibility to secondary infections (Almeida et al. 2019b, Kim et al. 2019, Kayani et al. 2021).

In the present study, the gut microbiome of the treatment group showed an almost complete shift of dominant genera, with the potentially probiotic *Lactobacillus* and *Weissella*, respectively decreasing by 6.5-fold and becoming absent in response to OTC. On the other hand, elements from the potentially pathogenic genera *Vibrio* increased by 6-fold and, surprisingly, *Photobacterium* spp. increased by 60-fold, when compared to the diseased (not yet treated) fish. This abundant *Photobacterium* likely corresponds to species other than *P. damselae* subsp. *piscicida*, since this was identified as the etiological agent of disease and presented susceptibility to OTC in antibiogram results. Instead, these strains could correspond to *P. damselae* subsp. *damselae* that has been frequently detected in the same fish farms as the *piscicida* subspecies, leading to visible enteric alterations. Importantly, there was also a 75-fold increase in Fusobacteria in the gut microbiome of gilthead seabream during antibiotic treatment, mainly leveraged by an increase in *Cetobacterium* sp. In a recent study, the expansion of *Cetobacterium somerae* in response to viral infection was suggested to increase the grass carp inflammatory reaction (Wu et al. 2020). Similar trends had already been detected in the gut of other fish treated with OTC (Nile tilapia and zebrafish; Zhou et al. 2018, Payne et al. 2021), rifampicin (mosquito fish; Carlson et al. 2017), and florfenicol (pacu; Sáenz et al. 2019). However, in the work by Payne et al. (2021), although Fusobacteria slightly increased after OTC treatment, they were already highly abundant prior to treatment, with this outcome suggested to be related to the resilience of the gut microbiome. Importantly, studies on *Vibrio* and *Cetobacterium* have shown that they can carry antibiotic resistance genes (ARGs), which could explain their increase in abundance during antibiotic treatments (e.g. Rocha et al. 2016, Sáenz et al. 2019).

Contrary to what occurred in the gill and gut microbiota, alpha-diversity tended to increase in the skin of seabream fingerlings in response to OTC treatment. This increase in diversity may have resulted from increased colonization from water microbiota, since previous works have shown that the skin shares a higher proportion of its microbiota with water, when compared to the gill microbiota (e.g. Rosado et al. 2021c). Moreover, this increase in diversity was concordant with an increase in enriched metabolic pathways, particularly the ones related to biosynthesis, which increased more than 2-fold during treatment. Furthermore, an increase of 30-fold in *Pseudoalteromonas* was observed in the skin, which agrees with results obtained in a previous study for the skin of the seabass after OTC administration (Rosado et al. 2019). A total of 39 % of the *Pseudoalteromonas* strains, including some isolated from gilthead seabream, produce antimicrobial metabolites (Pujalte et al. 2007). Thus, these and our results further support that these strains might play a key role in antimicrobial defense (Offret et al. 2016).

Overall, functional changes to the gut microbiome caused by OTC treatment were less severe when compared to skin and gill microbiomes. The number of enriched metabolic pathways in the gut of fish undergoing treatment remained stable and they were also generally concordant with the ones enriched in diseased fish. Nevertheless, it is worth noting that pathways related to biosynthesis in the skin and gut showed a shift toward the biosynthesis of vitamins, which are known to be essential for the host’s health (LeBlanc et al. 2013).

### Recovery of the mucosal microbiome of the gilthead seabream

Some studies evaluating the impact of OTC usage in fish microbiota have shown that, after administration ceases, recovery of the microbial communities to a state similar to what is observed in healthy individuals is not achieved. For example, adult seabass skin and gill microbial communities presented differences in taxonomic composition and structure, but not alpha-diversity, after a 2-week recovery (Rosado et al. 2019). However, in zebrafish, the gut microbiome was seen to show indications of recovery after 2 months of exposure to OTC treatment (Almeida et al. 2021). Overall, the present results indicate that there were persistent deleterious changes to the seabream microbiota following disease and antibiotic treatment, and that recovery to the initial diversity and predicted function did not occur even after almost a month.

Overall, there were differences in the dominant taxa observed in the Recovery2 group and the initial groups in all three tissues. Particularly, the bacterial composition changed significantly in the skin with potentially opportunistic pathogens such as *Psychrobacter* and *Vibrio* considerably increasing between Recovery1 and Recovery2 periods. The gut bacterial composition has also changed just after treatment (Recovery1), with an important increase of *Lactobacillus* and a decrease of *Cetobacterium*, genera known for their potentially probiotic role in the gut (Hoseinifar et al. 2018, Van Doan et al. 2019). These changes were accompanied by an increase of *Photobacterium*, which thus became the second most abundant bacteria in the gut, but was absent in the next sampling point (Recovery2). At this point, the gut bacterial community was again totally changed (dominated by *Catenococcus, Pseudomonas* and an unknown bacteria genus), and still different from the initial sampling points (Arrived and Asymptomatic).

More importantly, there were striking differences regarding the predicted metabolic pathways associated with the microbiota of Asymptomatic and Recovery2 groups, particularly for the gill microbiota where microbiome function was severely impaired, with only four metabolic pathways related to biosynthesis enriched at the end of sampling (vs. 127 predicted metabolic pathways enriched in the Asymptomatic group). Although to a lesser extent, a decrease in diversity of predicted metabolic pathways was also observed in the skin and gut microbiome of the seabream when comparing the Asymptomatic and Revovery2 groups, showing that function was generally affected. Importantly, 2 days after the end of antibiotic treatments (Recovery1) there were two enriched metabolic pathways related to antibiotic resistance in the skin most likely due to OTC treatment, which should raise concern since the skin seems to be enriched with potential pathogens.

In a previous study on European seabass, although the contribution of microbiota from water to the skin decreased drastically during periods of dysbiosis (see Fig. 4 from Rosado et al. 2021c), it increased in the months following. Although we have not measured the contribution of water microbiota to bacterial succession, it is likely that some of the differences in microbiome composition between asymptomatic and recovered fish could have resulted from the recruitment of bacteria from the surrounding water. Although fish mucosae are regarded as being highly selective, it seems that if such a recruitment process took place, it did not lead to functional recovery.

Fish microbiotas are highly dynamic and variable over time (e.g. Rosado et al. 2021a,c), and so temporal differences were expected to be found. Nevertheless, our results clearly disclose that disease and antibiotic treatment have an important impact on fish mucosa microbiotas, and that recovery of the mucosal microbiome after such events does not necessarily correspond to structure, composition and functional recovery.

## Conclusions

The microbiome of fish mucosal tissues plays a central role in fish homeostasis, being important in maintaining immunity. In the present study, we characterized dysbiosis occurring in the skin, gill, and gut of seabream juveniles during an infection outbreak caused by *P. damselae* subsp. *piscicida* and subsequent antibiotic treatment with OTC. Although the microbial response differed between tissues, overall differences in composition, diversity, structure, and predicted function were denoted in all mucosae. OTC also seemed to promote antimicrobial resistance, particularly in bacteria residing in the skin of seabream fingerlings, which also harbored several potential pathogens after the onset of the disease. It is important to notice that, even though there is good evidence that fish mucosae are highly selective and that the influence of the water microbiome is low (e.g. Leonard et al. 2014, Krotman et al. 2020), we cannot exclude the fact that the bacterial recruitment from the surrounding environment might play a role in shaping and succession of the fish microbiota after dysbiosis. Unfortunately, water microbial communities were not sampled during this study and thus this corroboration is not possible, but we stress the importance of doing it in future studies. Finally, changes observed between the microbiomes of fish just upon arrival, when mortality occurred, and fish already acclimated to the sea-cages point to an impact of transportation on fish microbiota, characterized by dysbiosis, including shifts in community diversity, structure, and composition, as well as function. Further studies including sampling points before and after transportation are needed to assess the impact of transportation on gilthead seabream microbiomes.

## Supplementary Material

xtad011_Supplemental_Files
