# Supplementary material for: Disruption of the skin, gill, and gut mucosae microbiome of gilthead seabream fingerlings after bacterial infection and antibiotic treatment
Source: FEMS Microbes. 2023 Jun 2;4:xtad011. doi: 10.1093/femsmc/xtad011 (PMC10306326; doi:10.1093/femsmc/xtad011)
Supplement: xtad011_Supplemental_Files [file xtad011_supplemental_files.zip › FEMSMC-2022-032 supplementary data - reviews and author response.docx]

**Reviews of manuscript FEMSMC-2022-032**

**Reviewer 1**

**Recommendation: Reject**

The paper by Rosado et al. is a well performed and interpreted study. However, its design has a major drawback which does not allow me to agree for its publication. The paper does not include any microbiota analysis of the surrounding water. While the water microbiota does not seem to overlap with the gut/skin/gill microbiota at least for fish of similar size with the one that the authors used in their study, the water microbiota can be an important player in the gill and skin microbiota shaping and succession, especially after some kind of perturbation, such as an infection; these tissues are in constant physical contact with a sea of microbes and are also the first tissues of the host to respond to any perturbation. The paper already includes several bacterial genera of seawater that are becoming abundant and have a free-living and/or attached way of living, but the paper cannot provide any information on whether these genera are abundant or not in the surrounding water, at which time point etc.

**Reviewer 2**

**Recommendation: Minor Revision**

This study reports overall changes in composition, diversity, structure and predicted functions in three distinct mucosae (gut, skin, gill) in gilthead seabream, sampled under different conditions.

The experimental design is very interesting: the authors anticipated that an outbreak of the disease caused by P. damselae ssp. piscicida would occur, and they took samples beforehand, during, and after the outbreak. This makes samples very interesting for comparison purposes, and provides a clear picture of what happens under natural conditions. This approach guarantees that the pathogen has entered the fish via its natural infection routes (whatever these may be), and eliminates the biass introduced when artificial, intraperitoneal infections are performed. It is also notable the monitoring of the microbiota after oxytetracycline treatment, an antibiotic largely used in fish farms against this pathogenic bacterium. This study detected a negative impact of antibiotic treatment in the microbiome, and alerts of an increase in gut samples, of Vibrio species after oxytetracycline treatment.

-Results section is tough Reading. The statistical analyses and their outputs are written in a fashion difficult to follow by the reader. It would be advisable to explain, after every asseveration of alpha-diversity and of other indexes (Faith’s, Shannon’s and so on), what the results actually mean. For example, when authors state that “a significant decrease in

alpha-diversity estimates occurred between Treatment and Recovery1 groups”, the reader would be relieved if authors say what does this actually mean. Comments like “this means that the overall diversity of genera or species bla bla bla...” or whatever.

Otherwise, the reader can be easily lost looking for what ecah index mean.

-In general, the results section looks not very clear to a potential reader. This is compensated by the discussion, that is quite informative. However, author should make an effort in re-writting parts of the Results section in order to make it more easily approachable by the readers.

-It is advised that the Results section is ended with a paragraph that states the Major changes in microbiota occurring in the Main transitions. I mean, what are the major changes upon infection by Pdp, and what are the major changes upon treatment with Oxytetracycline. This can be represented in a simple figure, a flow-diagram or something of the like. Otherwise the main info is difficult to grasp by the redaers.

-Line 30. Pathogen “infectious” should read pathogen “infections”, right?

-Lines 123-124: P. damselae ssp. piscicida (Pdp) was identified by API20E. Please, can you provide additional evidence of these isolates being Pdp? colony size? general appearance? Any plate-and-tube tests? Any subspecies specific PCR test?. API20E is more intended for clinical use, though it is also used for marine bacteria as well, but interpretation of results and final diagnosis need caution.

**Author's Response to Decision Letter for (FEMSMC-2022-032)**

Associate Editor's Comments to Authors:

If you are able to address the critiques of the 1st reviewer regarding the microbiota in the surrounding water, we would be happy to consider a revised manuscript.

A: We thank the Associate Editor for the opportunity to consider the revised manuscript. We agree with Reviewer 1 that the microbiome of the surrounding water would have enabled us to understand whether it has an important effect. However, the literature shows that the water influence on the microbiome of fish mucosae dynamics is limited. In the revised manuscript, we addressed this question both in the “Introduction”, “Discussion”, and “Conclusions” sections, and we hope it can now be accepted.

Reviewers' Comments to Authors:

Reviewer: 1

Comments to the Author

The paper by Rosado et al. is a well performed and interpreted study. However, its design has a major drawback which does not allow me to agree for its publication. The paper does not include any microbiota analysis of the surrounding water. While the water microbiota does not seem to overlap with the gut/skin/gill microbiota at least for fish of similar size with the one that the authors used in their study, the water microbiota can be an important player in the gill and skin microbiota shaping and succession, especially after some kind of perturbation, such as an infection; these tissues are in constant physical contact with a sea of microbes and are also the first tissues of the host to respond to any perturbation. The paper already includes several bacterial genera of seawater that are becoming abundant and have a free-living and/or attached way of living, but the paper cannot provide any information on whether these genera are abundant or not in the surrounding water, at which time point etc.

A: The reviewer is correct, and sampling water microbial communities would have helped clarify the effects of water microbiota in microbial succession after the disruption. However, we did not have that opportunity during this study. Nevertheless, despite the close contact of water and fish mucosae, the literature shows that the water influence is minor and, even though taxa are shared, the percentage reported is very low, possibly due to the fact that the fish tissues are highly selective and to a mucus barrier between skin/gill and water. The reviewer's comments on the role of water microbiota on bacterial suggestion after dysbiosis led us to look into the microbiome literature but we have found no studies testing the effects of water after dysbiosis on fish microbiota, although we admit we may have missed important papers. This comment also prompted us to look at our own data on European seabass published in 2021 (ref). In this paper, we monitored the skin and gill microbiota of healthy and untreated fish for 12 months, with sampling occurring every other week. In this time frame, we identified at least three dysbiotic events (April, July, and November) that occurred concomitantly in the gill and skin, which led to similarly important changes in regards to alpha diversity (which always had a significant decrease), beta-diversity and microbiome composition. In the analysis of this paper, we tested for each tissue (gill/skin) for each month (i.e. 2 sampling points merged) the contribution of water sampled during that month, the contribution of the other tissue sampled in that month (skin to gill/gill to skin) and the contribution of the same tissue using the diversity samples obtained in the previous month (this we interpreted as being the proportion of the microbiota that remained stable) (Fig 4 of that paper). The results showed high variation in the contribution of each source of microbiome tested, with unknown sources always predominant. We also saw that the skin frequently shared similarities with the water microbiome as opposed to the gill where the influence was usually limited. Although we have not discussed the effect of the water in microbial succession after dysbiosis in that paper we could now notice that the influence of water was almost null in the months where dysbiosis was observed, in fact, the decrease in the contribution of water to the skin was quite noticeable. However, for the skin where the contribution of water was noticeable, this contribution was resumed in the following months. In the gill microbiota, we could notice an increase in the water contribution in the two months after dysbiosis on only one occasion. In the framework of the present study, considering these previous observations and not excluding that water microbiome might play a role, we expect the role of water in the time frame where dysbiosis occurred in these tissues to have been negligible. Especially since important changes occurred both in the skin (more affected by water) and gill (comparatively less affected by water), and most times also in the gut. However, we do think this contribution might have increased during the recovery period, especially in the skin. Regarding this, we added information and references to the “Introduction” and discussed this potential effect on the recovery period in the “Discussion” and “Conclusion” sections. We think that the discussion is more interesting now that we have included water as a potential source for diversity increases in the skin, which we think is the most probable effect that the water may have had.

Reviewer: 2

Comments to the Author

This study reports overall changes in composition, diversity, structure and predicted functions in three distinct mucosae (gut, skin, gill) in gilthead seabream, sampled under different conditions.

The experimental design is very interesting: the authors anticipated that an outbreak of the disease caused by P. damselae ssp. piscicida would occur, and they took samples beforehand, during, and after the outbreak. This makes samples very interesting for comparison purposes, and provides a clear picture of what happens under natural conditions. This approach guarantees that the pathogen has entered the fish via its natural infection routes (whatever these may be), and eliminates the biass introduced when artificial, intraperitoneal infections are performed. It is also notable the monitoring of the microbiota after oxytetracycline treatment, an antibiotic largely used in fish farms against this pathogenic bacterium. This study detected a negative impact of antibiotic treatment in the microbiome, and alerts of an increase in gut samples, of Vibrio species after oxytetracycline treatment.

A: We thank the reviewer for this comment.

-Results section is tough Reading. The statistical analyses and their outputs are written in a fashion difficult to follow by the reader. It would be advisable to explain, after every asseveration of alpha-diversity and of other indexes (Faith’s, Shannon’s and so on), what the results actually mean. For example, when authors state that “a significant decrease in alpha-diversity estimates occurred between Treatment and Recovery1 groups”, the reader would be relieved if authors say what does this actually mean. Comments like “this means that the overall diversity of genera or species bla bla bla...” or whatever.

Otherwise, the reader can be easily lost looking for what ecah index mean.

A: We understand the results are difficult to read. Previously, we decided to organize this section by tissue, but considering the reviewer’s comments, we have decided to re-write them in chronological order. We hope now the “Results” section is easier to follow. We have also added some clarification about the indexes when they are first mentioned in the text.

-In general, the results section looks not very clear to a potential reader. This is compensated by the discussion, that is quite informative. However, author should make an effort in re-writting parts of the Results section in order to make it more easily approachable by the readers.

A: As mentioned in the previous comment, we have decided to rewrite the results in chronological order. We hope now the “Results” section is clearer.

-It is advised that the Results section is ended with a paragraph that states the Major changes in microbiota occurring in the Main transitions. I mean, what are the major changes upon infection by Pdp, and what are the major changes upon treatment with Oxytetracycline. This can be represented in a simple figure, a flow-diagram or something of the like. Otherwise the main info is difficult to grasp by the redaers.

A: As we have rewritten the “Results” section mentioning the results in chronological order, and due to the complexity of the results we did not add a section about the major changes occurring during the main transitions.

-Line 30. Pathogen “infectious” should read pathogen “infections”, right?

A: That is correct, we made the change.

-Lines 123-124: P. damselae ssp. piscicida (Pdp) was identified by API20E. Please, can you provide additional evidence of these isolates being Pdp? colony size? general appearance? Any plate-and-tube tests? Any subspecies specific PCR test?. API20E is more intended for clinical use, though it is also used for marine bacteria as well, but interpretation of results and final diagnosis need caution.

A: Further information was added to the “Materials and Methods” section, “Experimental design and sample collection” subsection: Results of the API 20E test showed small colonies with tiny dots morphology, streak growth, with no hemolysis, negative for gram coloration, and positive for catalase, oxidase, and motility. Additionally, using antibiogram disc diffusion, colonies were shown to be susceptible to oxytetracycline, with a disc diameter of 36 mm.

**Reviews of manuscript FEMSMC-2022-032.r1**

**Reviewer 2**

**Recommendation: Minor Revision**

The manuscript was been improved, as the authors have made an effort to convey a clearer message.

There is a point that deserve attention: In page 5, line 136, the authors describe Photobacterium damselae subsp. piscicida as "positive for catalase, oxidase, and motility".

This result is absolutely misleading. P. damselae subsp. piscicida is non motile. If the bacteria the authors have been studied is indeed motile, then it is necessary to completely modify the story being told here. Please, clarify this issue.

A minor point also, is that the authors cite a reference as "Jesús, 2002" (see page 15, line 475). This sounds weird, I understand the authors refer to "Romalde, 2002". Indeed the reference list, in page 22, lines 689-690, lists such reference in a wrong format. As far as I know, this author's name should be cited as Romalde, J.L (Romalde is a surname, whereas Jesus is a first name). Please correct the format of this reference.

**Reviewer 3**

**Recommendation: Accept**

In my opinion, the authors have made sufficient revisions to the manuscript based on previous comments to warrant acceptance for publication.
